# Alpha‐Actinin‐3 Deficiency Links Genetic Susceptibility to Renal Fibrosis: Evidence From Hemodialysis Patients and Murine Models

**DOI:** 10.1096/fj.202502803RRR

**Published:** 2026-02-12

**Authors:** Raisa B. Santos, Hellena Storch Vieira, Alice K. Scheer, Larissa R. Ribeiro, Cledia F. Silva, Rafael B. Orcy, Ines Schadock, Alexandre Budu, Ronaldo Carvalho Araujo, Augusto Schneider, André F. Rodrigues, Michael Bader, Maristela Böhlke, Carlos Castilho Barros

**Affiliations:** ^1^ Federal University of São Paulo São Paulo Brazil; ^2^ Laboratory of Nutrigenomics and Metabolism Federal University of Pelotas Pelotas Brazil; ^3^ Catholic University of Pelotas Pelotas Brazil; ^4^ Department of Experimental Toxicology German Federal Institute for Risk Assessment Berlin Germany; ^5^ Max Delbrück Center for Molecular Medicine Berlin Germany; ^6^ German Center for Cardiovascular Research (DZHK), partner Site Berlin Berlin Germany; ^7^ Charité Universitätsmedizin Berlin Corporate Member of Freie Universität Berlin and Humboldt‐Universität Zu Berlin Berlin Germany; ^8^ University of Lübeck Institute for Biology Lübeck Germany

**Keywords:** ACTN3, chronic kidney disease, fibrosis, gene expression, hemodialysis, rs1815739

## Abstract

The X allele of ACTN3 R577X polymorphism results in α‐actinin‐3 deficiency and has been associated with muscle damage and impaired recovery. While its role has been explored in musculoskeletal and cardiac contexts, no studies have evaluated its impact on chronic kidney disease (CKD). To investigate the prevalence of the ACTN3 R577X polymorphism in patients with end‐stage renal disease undergoing hemodialysis (HD) and explore its potential involvement in renal fibrosis through experimental models. A total of 217 HD patients and 413 healthy controls were genotyped for the ACTN3 R577X polymorphism. Associations with clinical variables were analyzed using multivariate regression. Renal Actn3 expression was evaluated in mice subjected to folic acid‐induced acute and chronic kidney injury. In vitro, fibroblasts were exposed to TGF‐β or LPS to assess gene expression responses. The X allele was significantly more frequent in HD patients (83.7% vs. 64.4%, *p* < 0.0001), and XX individuals began HD up to 11 years earlier than RR homozygotes. Experimental models showed persistent upregulation of Actn3 in fibrotic kidneys and in TGF‐β‐treated fibroblasts, but not in inflammatory conditions. Actn3 expression paralleled that of fibrosis markers such as Col1a1 and Acta2. The ACTN3 X allele is associated with earlier onset of renal failure and increased susceptibility to tubulointerstitial disease. Experimental data support its involvement in renal fibrosis. ACTN3 genotyping may help identify patients at greater risk for CKD progression.

## Graphical abstract

This study links the ACTN3 R577X polymorphism to chronic kidney disease (CKD), specifically in patients undergoing hemodialysis (HD). In parallel, increased Actn3 expression in murine models of kidney injury supports a functional role for ACTN3 in renal pathophysiology. AddiBonally, our findings in primary mouse embryonic fibroblasts reveal that Actn3 is upregulated in response to TGF‐β, a key profibroBc cytokine, thereby posiBoning ACTN3 as a potenBal modulator of the myofibroblast phenotype.

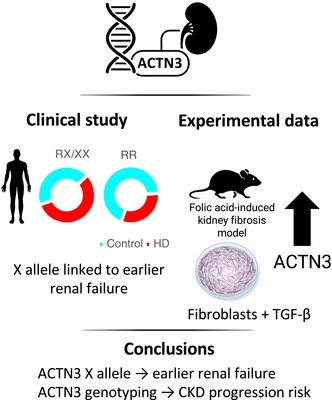

## 1 Introduction

Chronic kidney disease (CKD) affects approximately 10% of the global population and is associated with high mortality rates. End‐stage kidney disease (ESKD) is fatal without renal replacement therapy, such as dialysis or kidney transplantation. However, both treatments are costly and often inaccessible, particularly in low‐resource settings, leading to more than one million deaths annually from untreated kidney failure (KF) [1].

Renal fibrosis represents the final common pathway of virtually all forms of progressive CKD. It is characterized by excessive deposition of extracellular matrix (ECM) components, which disrupts normal kidney architecture and function, ultimately resulting in kidney failure [2]. The development of renal fibrosis is driven by complex molecular processes involving multiple cell types and signaling pathways [3, 4, 5].

Actinins are actin‐binding proteins that crosslink cytoskeletal filaments and are expressed in nearly all cell types. The human genome encodes four actinin isoforms (ACTN1–4), each with conserved structure and function. Mutations in these genes have been associated with various hereditary conditions [6]. Notably, mutations in the ACTN4 gene have been implicated in focal segmental glomerulosclerosis, a kidney disease that frequently leads to CKD and ESKD [7].

Alpha‐actinin‐3 (ACTN3), encoded by the ACTN3 gene, is predominantly expressed in type II (fast‐twitch) skeletal muscle fibers and plays a key role in muscle contraction and structural support [8]. Growing evidence suggests that ACTN3 may also participate in tissue remodeling and fibrosis in various organs [9].

ACTN3 is expressed in non‐muscle cell types, including ascending limb cells of Henle's loop, fibroblasts, lymphocytes, and leukocytes [10]. The ACTN3 gene harbors a common nonsense polymorphism, R577X (rs1815739), which replaces an arginine codon with a premature stop codon, resulting in complete loss of ACTN3 protein in homozygous individuals (XX genotype) [11]. Approximately 16% of the global population is homozygous for the X allele. ACTN3 deficiency has been associated with altered muscle function, impaired adaptation to mechanical stress, and increased susceptibility to muscle damage, which may promote fibrotic remodeling [12, 13]. Recent studies have linked the ACTN3 R577X polymorphism to exertional rhabdomyolysis in athletes—an acute muscle injury that can lead to acute kidney injury (AKI) [13, 14, 15]. The absence of ACTN3 appears to impair muscle regeneration, but its influence on fibrogenic signaling remains unclear.

ACTN3 has also been implicated in cardiac fibrosis. Its deficiency has been associated with maladaptive remodeling and increased fibrotic response in cardiomyocytes under stress [9]. This has been attributed to the activation of profibrotic pathways such as those involving transforming growth factor‐beta (TGF‐β) and angiotensin II, which stimulate fibroblast activation and ECM deposition [16]. Although direct evidence linking ACTN3 to fibrosis in the kidney is limited, its role in maintaining cytoskeletal integrity and regulating mechanotransduction suggests it may contribute to fibrotic processes in multiple organs, including the kidney [17, 18]. Furthermore, although collagen overdeposition is well known as a damaging process, a study has already shown that collagen I plays a role in recovery processes in certain types of kidney diseases.

Despite its expression in tubular cells of the ascending limb of Henle's loop and in renal fibroblasts [10], the ACTN3 R577X polymorphism has never been studied in patients undergoing chronic hemodialysis. Understanding whether this polymorphism contributes to kidney disease progression could offer new insights into fibrotic mechanisms and genetic susceptibility in CKD.

Based on this, the aim of the present study was to evaluate the prevalence of the ACTN3 R577X polymorphism in patients with end‐stage renal disease on hemodialysis and investigate its association with clinical parameters. Additionally, we explored ACTN3 gene expression and its relationship with fibrosis in experimental models of kidney injury.

## 2 Methods

### 2.1 Human Study

This cross‐sectional observational study involved genotyping of the R577X polymorphism in the ACTN3 gene in 217 patients with CKD undergoing hemodialysis (HD) at São Francisco de Paula University Hospital (Pelotas, Brazil), and 413 healthy individuals residing in the same geographic area. The study was approved by the local ethics committee (CAAE 64821816.0.0000.5339; approval no. 1.940.521), and all participants provided written informed consent prior to enrollment. Genotyping was performed in duplicate by two independent researchers to ensure accuracy. Inclusion criteria for the CKD group were: age ≥ 18 years and undergoing HD treatment for at least 3 months. Hemodialysis consisted of 4‐h sessions, three times per week, using Fresenius 4008‐S machines (Fresenius, Bad Homburg, Germany) and low‐flux polysulfone dialyzers. Sociodemographic and clinical data were obtained from medical records. Albuminuria was not analyzed because most hemodialysis patients had negligible or absent urine output due to end‐stage renal disease, preventing reliable quantification.

### 2.2 Genotyping

Genomic DNA was extracted from peripheral blood samples according to previous protocol [19].

Allele‐specific amplification of the ACTN3 R577X polymorphism was performed using the amplification refractory mutation system polymerase chain reaction (ARMS‐PCR). The four primers used are described in Table 1. The protocol described by Schadock et al. [19] was followed, using 2× GoTaq Green Master Mix (Promega, Cat. M7122). Each sample was independently genotyped by two different researchers, and results were considered valid only when a consensus was reached. The accuracy of the ARMS‐PCR method was validated by comparison with a fluorescence‐based real‐time PCR assay using the commercially available TaqMan SNP Genotyping Assay (Assay ID: C_590093_1, Applied Biosystems, Foster City, CA, USA). The assay included allele‐specific probes and flanking primers and was run using a premixed PCR master mix containing AmpliTaq DNA Polymerase Gold (Applied Biosystems) in a 20 μL total reaction volume. PCR conditions included an initial denaturation at 95°C for 10 min, followed by 50 cycles of 15 s at 95°C and 1 min at 60°C. Reactions were performed using an ABI 7500 Real‐Time PCR System (Applied Biosystems). The comparison revealed 100% concordance between the ARMS‐PCR and TaqMan methods.

**TABLE 1:** Sequences of the fragment‐specific external primers and allele‐specific internal primers used for genotyping by PCR.

Name	Sequence	Product size
hACTN3f	5′‐CGCCCTTCAACAACTGGCTGGA‐3′	690 bp with hACTN3r
hACTN3r	5′‐GATGAGCCCGAGACAGGCAAGG‐3′	
hACTN3Tif	5′‐CAACACTGCCCGAGGCTGACTG‐3′	318 bp with hACTN3r
hACTN3Cir	5′‐CATGATGGCACCTCGCTCTCGG‐3′	413 bp with hACTN3f

### 2.3 Mice and In Vitro Experiments

In addition to the human genetic component, experimental models were used to explore gene expression alterations related to renal fibrosis, through in vivo studies in mice and in vitro experiments with mouse embryonic fibroblasts (MEFs).

### 2.4 Effects of Acute Kidney Injury on Renal Actn3 Expression

All animal procedures were conducted in accordance with institutional guidelines and were approved by the Ethics Committee on Animal Use (CEUA) of the Federal University of São Paulo (UNIFESP). Male C57BL/6 mice (9–12 weeks old, 20–27 g) were obtained from the Animal Care Facility of UNIFESP. Animals were housed in standard cages with ad libitum access to food and water. A total of 6–8 animals per group were used.

To investigate the effects of inflammation on renal *Actn3* mRNA expression, acute kidney injury was induced by a single intraperitoneal (i.p.) injection of folic acid (240 mg/kg; Sigma‐Aldrich, St. Louis, MO, USA) dissolved in 0.3 M sodium bicarbonate (NaHCO_3_). Control mice received an equivalent volume of vehicle (0.3 M NaHCO_3_) via the same route.

We also analyzed fibrosis by histology. Kidney samples were processed by an external laboratory and stained with picrosirius red to analyze collagen deposition. The images were analyzed using ImageJ and FIJI software [20].

An independent renal fibrosis dataset was generated using angiotensinogen‐deficient mice (AGT‐KO), a model characterized by progressive interstitial fibrosis [21]. Kidneys from this model were collected at 12 weeks of age, and mRNA levels of Actn3, Col1a1, Fn1, and Tgfb1 were measured by RT‐qPCR. These animals have been shown to exhibit fibrotic remodeling in the renal interstitium [21]. Gene expression changes in AGT‐KO kidneys were compared to wild‐type (WT) littermates.

### 2.5 Blood Sampling and Tissue Collection

Mice were euthanized at 7‐ and 28‐days post‐injection. Prior to euthanasia, animals were anesthetized with a mixture of ketamine (91 mg/kg) and xylazine (9.1 mg/kg), administered intraperitoneally. Blood was collected via cardiac puncture and centrifuged at 2000 × *g* for 20 min. Plasma was stored at −20°C for subsequent analysis. Kidneys were excised, decapsulated, and transversely sectioned. Tissues were immediately frozen in liquid nitrogen and stored at −80°C until further processing.

### 2.6 Assessment of Renal Injury

Serum urea and creatinine levels were used as a biochemical marker of renal injury and function on Days 7 and 28 following folic acid administration. Quantification was performed using a commercially available colorimetric assay kit (Labtest, Lagoa Santa, Brazil), according to the manufacturer's instructions.

### 2.7 In Vitro Cell Culture

Mouse embryonic fibroblasts (MEFs) were isolated from C57BL/6 mice and cultured at 37°C in a humidified incubator with 5% CO_2_. Cells were maintained in Dulbecco's Modified Eagle Medium (DMEM) with GlutaMAX, high glucose, and pyruvate, supplemented with 10% fetal bovine serum (FBS) [22].

For experiments, MEFs were seeded into 48‐well plates and subjected to two treatment protocols. To induce an inflammatory response, cells were treated with lipopolysaccharide (LPS; 2 μg/mL) for 24 h, with all conditions performed in triplicate [23]. To promote myofibroblast differentiation, cells were treated with transforming growth factor beta (TGF‐β; 10 ng/mL) for 24 h [24].

### 2.8 Real‐Time PCR

Total RNA was extracted using the TRIzol reagent protocol (Invitrogen, Carlsbad, CA, USA), following the manufacturer's instructions. Complementary DNA (cDNA) was synthesized using the High‐Capacity cDNA Reverse Transcription Kit (Applied Biosystems, Waltham, MA, USA). Standard curves were generated to determine the amplification efficiency for each primer pair. Quantitative real‐time PCR (qPCR) was performed using a SYBR Green system (Thermo Scientific, Waltham, MA, USA) with specific primers. Primers were designed using the Primer3 web tool, and their specificity was verified using NCBI Primer‐BLAST. PCR cycling conditions were: initial denaturation at 95°C for 10 min, followed by 45 cycles of 30 s at 95°C, 30 s at 60°C, and 30 s at 72°C. Target mRNA expression was normalized to 18S rRNA and analyzed using the comparative threshold cycle (*Ct*) method (2^−ΔΔ*Ct*^). Gene expression levels were normalized to the vehicle‐treated control group and expressed as fold change.

### 2.9 Amplicon Sequence Analysis

To confirm the specificity of the qPCR products, amplicons were first verified by melting curve analysis and agarose gel electrophoresis (2% agarose). PCR products were purified, then submitted for Sanger sequencing (Exxtend LTDA, Campinas, Brazil). The resulting sequences were aligned using the BLAT tool against the NCBI nucleotide database, confirming that the amplicons corresponded to the expected *Actn3* gene fragment.

### 2.10 Statistical Analysis

Multilevel mixed‐effects regression followed by treatment‐effects estimation was used to compare the number of X alleles and associated clinical variables. The Shapiro–Wilk test and the “ladder of powers” command in STATA were applied to assess data normality and identify the most appropriate transformation for each dataset. All regression models were adjusted for sex and ethnicity.

Genotype frequencies between patients and healthy individuals from the same geographic area were compared using contingency table analysis in GraphPad Prism (GraphPad Software, San Diego, CA, USA). Logistic regression was used for binary outcomes.

Data from animal and cell‐based experiments were analyzed using an unpaired *t*‐test. All datasets were tested for normal distribution prior to statistical analysis. A *p*‐value equal or lower than 0.05 was considered significant.

## 3 Results

### 3.1 Human Sample Characterization

Two hundred and seventeen HD patients agreed to participate in the study, and 184 of them authorized the sample collection for genotyping. Another 413 healthy control individuals living in the same neighborhood as the patients were genotyped. Patients who declared themselves white constituted the majority (79.4%), and 43.3% were women. The mean age at admission to HD was 54.0 ± 17.9 and 57.6 ± 16.7 years for women and men, respectively. Approximately half of the participants had hypertension and diabetes (Table 2). There was no difference between the frequency of race and gender between patients and the control group.

**TABLE 2:** Sample characterization of HD patients and the healthy control group.

	Unit	Patients		Total	Controls		Total
		Gender			Gender		
		Female	Male		Female	Male	
Participants	% (*n*)	43.3 (94)	56.7 (123)	217	54.7 (226)	45.3 (187)	413
Ethnicity							
White	% (*n*)	40.8 (71)	59.2 (103)	174	50.8 (158)	49.2 (153)	311
Black	% (*n*)	53.5 (23)	46.5 (20)	43	54.9 (56)	45.1 (46)	102
Age	Mean ± SD	58.0 ± 17.2	58.5 ± 16.2		34.1 ± 28.1	36.0 ± 21.9	
Age in HD	Mean ± SD	54.0 ± 17.9	57.6 ± 16.7		—	—	
AH	% (*n*)	40.0 (58)	60.0 (87)	145	—	—	
Diabetes	% (*n*)	42.1 (37)	57.9 (51)	88	—	—	

### 3.2 The Prevalence of X Allele Is Higher in Patients Than in Controls

The prevalence of the XX genotype was twice as high in patients on hemodialysis (31.52% vs. 17.92%, *p* = 0.0002), compared to the control group. The X allelic frequency was almost 50% higher in those patients (83.69 vs. 64.41%, *p* < 0.0001) (Table 3). Allelic distribution both in patients and controls was balanced according to Hardy–Weinberg analysis.

**TABLE 3:** ACTN3‐R577X genotype distribution and HWE analyses.

Genotype	Hemodialysis		Control		
	Observed	Expected	Observed	Expected	Contingency table
	% (*n*)	% (*n*)	% (*n*)	% (*n*)	*p*
ACTN3‐R577X					
RR	16.3 (30)	18.0 (33.1)	35.6 (147)	35.6 (143.0)	< 0.0001
RX	52.1 (96)	48.9 (89.9)	46.5 (192)	48.4 (200.0)	
XX	31.5 (58)	33.2 (61.1)	17.9 (74)	16.9 (70.0)	
X Allele frequency	0.576		0.412		
HWA	*p* = 0.355		*p* = 0.414		

Abbreviations: ACTN3‐R577X, alpha‐actinin‐3 R577X polymorphism; HWA, analysis for Hard‐Weinberg equilibrium.

### 3.3 Allele X Is Associated With Early Indication of HD Treatment in Patients With CKD

Carriers of the X allele started HD up to 11 years earlier compared to patients with the RR genotype (average age starting HD treatment: XX, 51.5 ± 2.14 years old vs. RR, 62.9 ± 2.49, *p* = 0.022). Heterozygous subjects started HD only 1 year later than those carrying the XX genotype (Table 4). Although patients with the X allele developed renal failure earlier, there was no influence of the R577X polymorphism on the survival of patients on HD after starting treatment, indicating that although there is a higher predisposition to chronic renal failure, there is no difference in the prognosis between genotypes after initiating the HD treatment. Clinical parameters, adjusted for gender and race are shown in Table 4. Dry weight, serum creatinine, post‐dialysis urea and parathyroid hormone (PTH) were not different between genotypes, but only related to gender and skin color as verified in *post hoc* tests.

**TABLE 4:** Clinical parameters of HD patients.

Variable	Unit	Actn3			
		RR	RX	XX	*p*
Age in HD	Years	62.9 ± 2.49	52.3 ± 1.76	51.5 ± 2.14	**0.0222**
Time in HD until obit	sqrt (years)	1.80 ± 0.18	2.08 ± 0.16	1.80 ± 0.20	0.1363
Dry weight	sqrt (g)	262.2 ± 4.41	260.73 ± 3.27	61.17 ± 4.42	0.0444*
Initial SAP	mmHg	144.5 ± 3.88	142.53 ± 2.30	143.04 ± 2.97	0.8699
Initial DAP	mmHg	83.8 ± 2.69	82.27 ± 1.49	84.34 ± 2.05	0.6063
Ultrafiltration	sqrt (uf)	47.3 ± 2.22	50.0 ± 1.07	50.1 ± 1.76	0.2706
Final SAP	mmHg	140.8 ± 3.46	135.5 ± 2.14	140.3 ± 2.63	0.4661
Final DAP	mmHg	81.0 ± 2.71	82.6 ± 1.31	81.3 ± 1.45	0.9479
Calcium	log (mg/dL)	2.20 ± 0.14	2.19 ± 0.010	2.20 ± 0.011	0.3807
Creatinine	sqrt (mg/dL)	2.72 ± 0.96	2.93 ± 0.044	2.91 ± 0.095	0.0003*
Phosphorus	mg/dL	4.46 ± 0.219	5.04 ± 0.182	4.94 ± 0.182	0.4754
Hematocrit	%	31.9 ± 1.05	33.4 ± 0.41	32.8 ± 0.85	0.188
Hemoglobin	sqrt (g/dL)	3.13 ± 0.056	3.22 ± 0.028	3.19 ± 0.042	0.0575
Potassium	log (mmol/L)	1.65 ± 0.029	1.64 ± 0.017	1.65 ± 0.021	0.8809
Sodium	cubic (mmol/L) 0.10^−6^	2.63 ± 0.037	2.65 ± 0.020	2.64 ± 0.032	0.3278
GPT	log (U/L)	2.32 ± 0.102	2.32 ± 0.053	2.37 ± 0.057	0.3396
Urea	sqrt (mg/dL)	10.7 ± 0.281	10.7 ± 0.267	10.9 ± 0.202	0.6806
Post dialysis urea	sqrt (mg/dL)	5.48 ± 0.229	5.95 ± 0.137	6.00 ± 0.208	0.0232*
PTH	log (pg/dL)	5.65 ± 0.175	5.84 ± 0.096	5.57 ± 0.147	0.0273
Albumin	cubic (g/dL)	71.9 ± 4.64	71.1 ± 2.47	70.9 ± 3.30	0.0569

*Note:* Mixed‐effects ML regression (*p* value) followed by treatment‐effects estimation in STATA program. Values presented as estimated outcome means for each genotype. After Shapiro‐Wilk normality test, it was used “ladder of powers” command in STATA to find the best fit method. Bold *p* values indicate significance after gender correction. * indicates gender effect.

* *p* value influenced by sex variable.

### 3.4 Allele X Is Associated With Tubulointerstitial Disease

Primary kidney diseases (PKD) causing KF were retrieved from the medical records and grouped by similarity of cause. An association with the X allele was observed in patients with PKD normally linked to tubulointerstitial involvement (*p* = 0.035, 95% CI 0.133527 to 1.545219). The PKDs considered as associated with tubulointerstitial disease was extracted from reviews [25, 26]. On the other hand, when considering only this parameter, it was not possible to demonstrate an association between any genotype and the nephron structure of the initial lesions in PKDs (*p* = 0.4181, 95% CI −0.6515916 to 0.6011207). Table 5 shows the genotypic frequency of each underlying disease and for the grouped diagnoses showing the association of X allele to PKD typically associated with tubulointerstitial disease.

**TABLE 5:** Primary kidney diseases leading to kidney failure and ACTN3‐R577X genotyping.

Grouped diagnostic	Primary kidney disease	*n*	Genotype %			Increased RX + XX frequency^a^	Starting kidney injury structure^b^	Primary diagnostic associated to tubulointerstitial disease^c^
			RR	RX	XX			
Polycystic kidney	Adult type polycystic kidney	15	13.3	40	46.7	Yes	Tubules	Yes
	Subtotal	15	13.3	40	46.7			
Obstructive	Medullary cyst of the kidney	1	0	100	0	Yes	Tubules	Yes
	Tuberous sclerosis	1	0	100	0	Yes	Tubules	Yes
	Multiple congenital malformations not classified elsewhere	1	0	100	0	Yes	Tubules	Yes
	Other hydronephrosis and unspecified	1	0	100	0	Yes	Tubules	Yes
	Tuberculosis sequelae of airways and unspecified organs	1	0	100	0	Yes	Tubules	Yes
	Twisting and narrowing of the ureter without hydronephrosis	1	0	0	100	Yes	Tubules	Yes
	Vesicoureteral reflux	24	8.3	50	41.7	Yes	Tubules	Yes
	Subtotal	30	6.7	56.7	36.7			
Ischemic nephropathy	Atherosclerosis of the renal artery	9	22.2	55.6	22.2	No	Tubules	Yes
	Subtotal	9	22.2	55.6	22.2			
Glomerulopathies	Amyloidosis	1	0	100	0	Yes	Glomerulus	Yes
	Glomerulopathies in HIV patients	3	33.3	33.3	3.33	No	Glomerulus	Yes
	Hypertensive kidney disease with kidney failure	2	0	100	0	Yes	Glomerulus	Yes
	Lupus erythematosus	2	0	100	0	Yes	Glomerulus	Yes
	Angiopathy	3	33.3	33.3	3.33	No	Glomerulus	Yes
	Multiple myeloma	2	0	50	50	Yes	Glomerulus	Yes
	Glomerulonephritis	16	12.5	50	37.5	Yes	Glomerulus	Yes
	Nephropathy induced by other drugs and biological substances	1	0	0	100	Yes	Glomerulus	Yes
	Recurrent and persistent haematuria	2	50	50	0	No	Glomerulus	No
	Nephrotic syndrome	4	25	50	25	No	Glomerulus	No
	Glomerular disorders in systemic connective tissue diseases	1	0	100	0	No	Glomerulus	No
	Subtotal	36	16.7	55.6	28.8			
Diabetic kidney disease	Glomerular disorders in diabetes mellitus	48	18.7	54.2	27.1	No	Glomerulus	No
	Subtotal	48	18.7	54.2	27.1			
Unknown Primary Disease	Acute renal failure with tubular necrosis	1	100	0	0	No	Unknown	Yes
	Chronic kidney failure unspecified	5	20	80	0	No	Unknown	No
	Unspecified contracted kidney	34	17.6	41.2	41.2	No	Unknown	Yes
	Other chronic kidney failure	2	0	100	0	Yes	Unknown	No
	Subtotal	43	18.6	46.5	34.9			
	Total	181	16	51.9	32			

^a^ Dichotomous variable indicating an increase in the frequency of the RX + XX genotypes comparing to the total genotypic distribution.

^b^ Categorical variable indicating initial nephron structure affected in primary disease.

^c^ Literature‐based dichotomous variable indicating whether the primary disease is frequently associated to interstitial tubule disease.

### 3.5 Renal Actn3 Gene Expression Is Upregulated 7 Days After Folic Acid‐Induced Acute Kidney Injury in Mice

**FIGURE 1:**
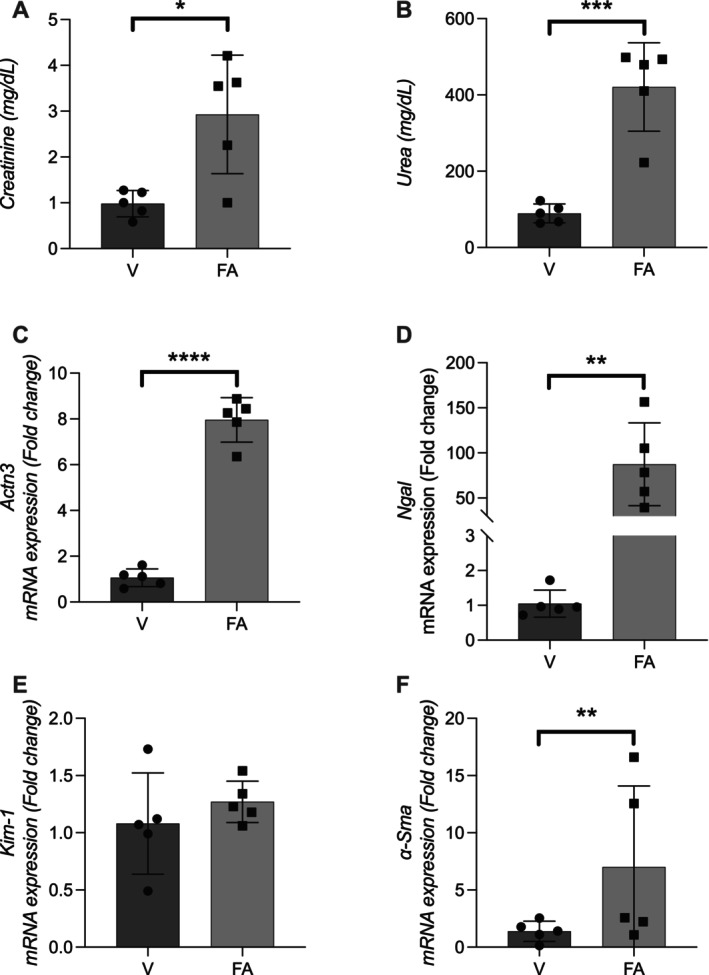
Renal gene expression of *Actn3* and markers of kidney injury and fibrosis activation 7 days after folic acid‐induced acute kidney injury in mice. Male C57BL/6J mice were administered a single intraperitoneal injection of folic acid (240 mg/kg) to induce acute kidney injury. After 7 days, plasma and kidney samples were collected for molecular analyses. (A) Plasma creatinine levels; (B) Plasma urea levels; (C–F) Renal mRNA expression levels of *Actn3, Lcn2* (*Ngal*), *Havcr1* (*Kim‐1*), and *Acta2* (*α‐SMA*), respectively. Data are presented as mean ± SEM. FA, folic acid group; V, vehicle group. Statistical comparisons were performed using an unpaired *t*‐test. * *p* < 0.05, **p < 0.01, ***p < 0.001, ****p < 0.0001.

Seven days after induction of acute kidney injury (AKI) by intraperitoneal injection of folic acid (240 mg/kg), mice exhibited marked renal dysfunction, as evidenced by significant increases in plasma creatinine (*p* < 0.05) and urea levels (*p* < 0.001) compared to vehicle‐treated controls (Figure 1A,B).

Renal mRNA expression of *Actn3* was significantly upregulated in the folic acid group, showing an approximately eightfold increase (*p* < 0.0001) (Figure 1C). Expression of Lipocalin‐2 (*Lcn2* or *Ngal*), a sensitive marker of kidney injury and mediator of fibrotic responses, was also significantly elevated (*p* < 0.01) (Figure 1D).

In contrast, no significant difference was observed in the expression of Hepatitis A Virus Cellular Receptor 1 (*Havcr1* or *Kim‐1*), another AKI marker, between groups (Figure 1E). Expression of Actin Alpha 2, Smooth Muscle (*Acta2* or *α‐SMA*), a myofibroblast differentiation marker, was significantly increased in the folic acid‐treated group (*p* < 0.01), suggesting activation of profibrotic remodeling processes (Figure 1F).

Together, these findings confirm successful induction of AKI and indicate that *Actn3* upregulation may be associated with early molecular events in kidney injury and fibrosis.

### 3.6 Renal Actn3 and Col1a1 Expression Remain Elevated 28 Days After Acute Kidney Injury in Mice

**FIGURE 2:**
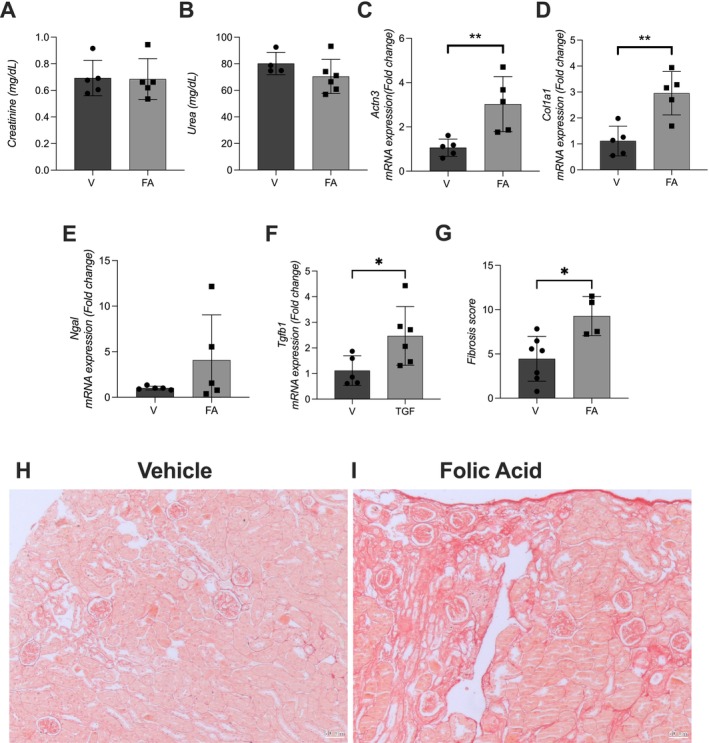
Long‐term renal expression of *Actn3*, fibrosis, and injury markers following folic acid‐induced kidney damage in mice. Male C57BL/6J mice received a single intraperitoneal injection of folic acid (240 mg/kg) to induce acute kidney injury. 28 days post‐injection, plasma and kidney samples were collected for analysis. (A) Plasma creatinine levels; (B) Plasma urea levels; (C–F) Renal mRNA expression levels of *Actn3*, *Col1a1* (collagen type I alpha 1 chain), *Lcn2* (also known as *Ngal*), and Tgfb1, respectively; (G) Fibrosis scores; (H and I) Picrosirius red‐stained sections to analyze collagen deposition. Data are presented as mean ± SEM. FA, folic acid group; V, vehicle group. Statistical analysis was performed using an unpaired *t*‐test. * *p* < 0.05, **p < 0.01.

Twenty‐eight days after induction of AKI via intraperitoneal injection of folic acid (240 mg/kg), plasma creatinine and urea levels returned to baseline, indicating recovery of renal function (Figure 2A,B). However, renal expression of *Actn3* remained significantly elevated in the folic acid‐treated group compared to vehicle controls (*p* < 0.01), suggesting that *Actn3* upregulation persists during the chronic phase of kidney injury (Figure 2C).

Similarly, expression of *Col1a1* (Collagen Type I Alpha 1 chain), a marker of fibrosis, was significantly increased in the folic acid group (*p* < 0.01) (Figure 2D), and this was accompanied by increased Tgfb1 expression (*p* < 0.05), indicating ongoing fibrotic activity. No statistically significant difference was observed in *Lcn2* (*Ngal*) expression between groups, although a trend toward elevation was noted in the folic acid group, with considerable inter‐individual variability (Figure 2E).

These findings suggest that, despite apparent recovery of renal function, molecular markers of fibrosis and cytoskeletal remodeling remain upregulated, supporting the presence of subclinical or ongoing chronic kidney damage.

**FIGURE 3:**
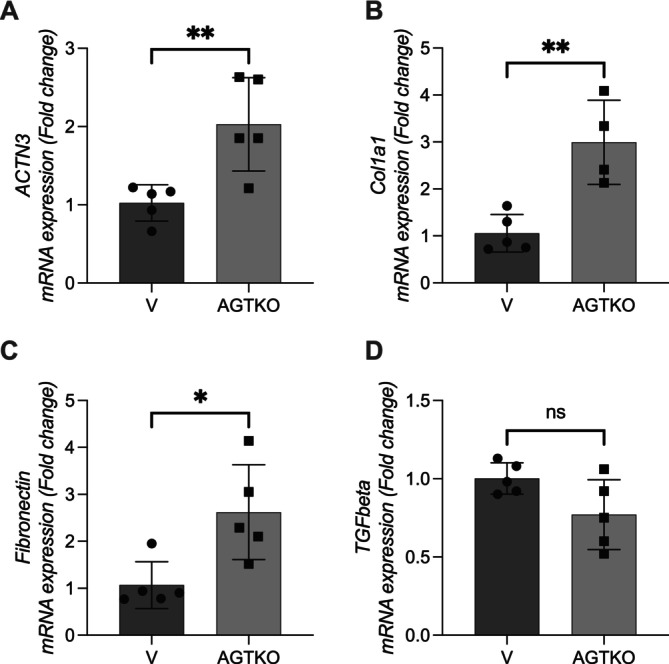
Renal expression of *Actn3*, fibrosis, and injury markers in knockouts for angiotensinogen (AGT‐KO). Renal RNA from male FVB/N (“V”; FVB/NCrl; Charles River Laboratories #207, RRID: IMSR_CRL:207) and angiotensinogen‐deficient mice [21] were analyzed for gene expression of: (A) Actn3; (B) Col1a1; (C) fibronectin and (D) TGF‐β. Data are presented as mean ± SEM. AGTKO, angiotensinogen knockout mice; V, wild‐type mice. Statistical analysis was performed using an unpaired *t*‐test. * *p* < 0.05, **p < 0.01.

The Angiotensinogen deficient mice (AGT‐KO) model showed marked upregulation of Actn3 expression in fibrotic kidneys, mirroring the findings observed in the folic acid model. Fibrosis‐related markers (Col1a1, Fn1) were also significantly elevated in AGT‐KO renal tissue, supporting a fibrogenic transcriptional profile. These results, presented in Figure 3, confirm that Actn3 activation is not restricted to folic acid nephropathy and occurs across independent fibrosis models.

### 3.7 TGF‐β Induces Myofibroblast Marker Expression and Upregulates Actn3 in Primary Renal Fibroblasts

To evaluate whether *Actn3* expression is modulated during myofibroblast transformation, primary mouse renal fibroblasts were treated with transforming growth factor beta (TGF‐β), and mRNA levels of fibrotic and cytoskeletal markers were assessed by quantitative PCR.

**FIGURE 4:**
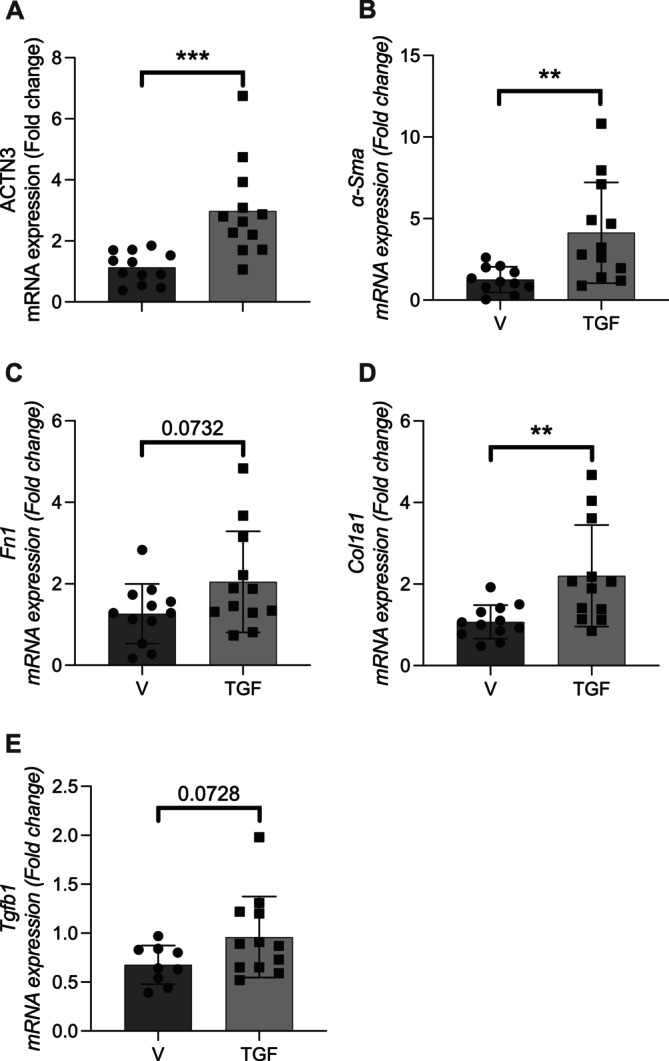
Gene expression analysis in cells undergoing myofibroblast transformation induced by TGF‐β treatment. Primary mouse renal fibroblasts were subjected to a myofibroblast transformation protocol using transforming growth factor beta (TGF‐β). Gene expression was assessed by quantitative PCR. TGF, TGF‐β‐treated cells; V, vehicle‐treated control cells. Data are presented as mean ± SEM. Statistical comparisons were performed using an unpaired *t*‐test. **p < 0.01, ***p < 0.001.

TGF‐β treatment significantly increased *Actn3* expression compared to vehicle‐treated controls (*p* < 0.001) (Figure 4A), suggesting that ACTN3 may be involved in the myofibroblast phenotype. As expected, TGF‐β also induced a robust upregulation of *Acta2* (*α‐SMA*), a canonical myofibroblast marker (*p* < 0.01) (Figure 4B).

Expression of *Col1a1* was also significantly elevated in TGF‐β‐treated cells (*p* < 0.01) (Figure 4D), consistent with activation of the fibrotic program. Although not statistically significant, there was a trend toward increased expression of fibronectin (*Fn1*) (*p* = 0.0732) and TGF‐β (*Tgfb1*) (*p* = 0.0728) in response to TGF‐β treatment (Figure 4C,E, respectively).

Collectively, these findings indicate that TGF‐β stimulates a fibrogenic transcriptional program in renal fibroblasts, accompanied by upregulation of *Actn3*, implicating this gene in the cellular response to profibrotic stimuli.

### 3.8 LPS Treatment Modulates the Expression of Inflammatory and Fibrotic Genes in Primary Renal Fibroblasts

**FIGURE 5:**
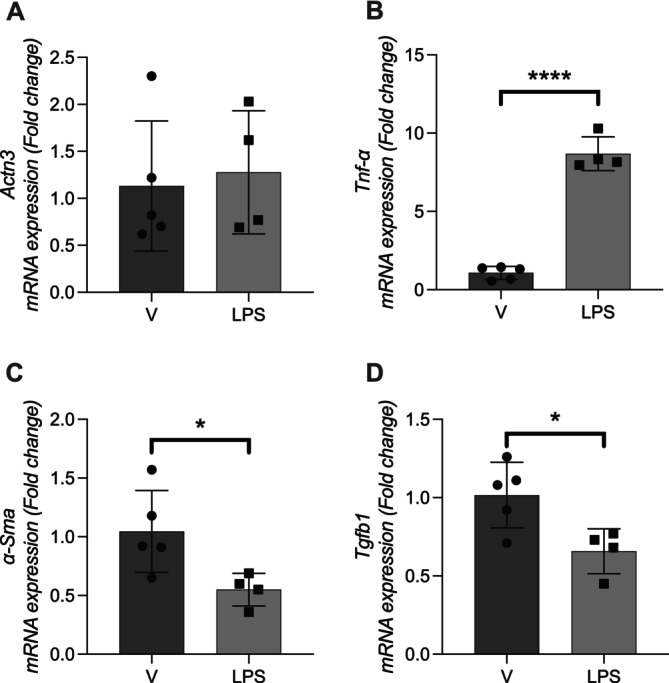
Gene expression analysis in fibroblast cells after LPS treatment. Primary mouse renal fibroblasts were subjected to LPS treatment. Gene expression was assessed by quantitative PCR. LPS, LPS‐treated cells; V, vehicle‐treated control cells. Data are presented as mean ± SEM. Statistical comparisons were performed using an unpaired *t*‐test. *p < 0.05, ****p < 0.0001.

To investigate the impact of inflammatory stimuli on gene expression, primary mouse embryonic fibroblasts were treated with lipopolysaccharide (LPS), and mRNA levels of key genes were quantified by RT‐qPCR. There was no significant change in *Actn3* expression following LPS treatment (*p* > 0.05), suggesting that this gene is not responsive to acute inflammatory activation (Figure 5A). In contrast, *Tnf* (tumor necrosis factor alpha) expression was markedly upregulated (*p* < 0.0001), confirming the effectiveness of the LPS stimulus in triggering an inflammatory response (Figure 5B).

Interestingly, *Acta2* (*α‐SMA*) expression was significantly downregulated in LPS‐treated cells compared to controls (*p* < 0.05), suggesting a reduction in the myofibroblast phenotype (Figure 5C). Similarly, *Tgfb1* expression was also significantly reduced (Figure 5D) (*p* < 0.05), indicating suppression of fibrotic signaling pathways in response to LPS. These findings suggest that while LPS strongly induces pro‐inflammatory signaling in fibroblasts, it may concurrently attenuate fibrotic gene programs, including *Tgfb1* and *Acta2*, but no changes are observed in *Actn3* expression.

## 4 Discussion

This study provides novel evidence linking the ACTN3 R577X polymorphism to chronic kidney disease (CKD), specifically in patients undergoing hemodialysis (HD). The significantly higher frequency of the X allele in HD patients, its association with earlier onset of dialysis, and the parallel increase in *Actn3* expression in murine models of kidney injury support a functional role for ACTN3 in renal pathophysiology.

The finding that the XX genotype is approximately twice as frequent in patients with CKD compared to healthy controls strongly suggests a genetic predisposition. Furthermore, individuals carrying the X allele required HD initiation up to 11 years earlier than RR individuals, supporting its clinical relevance. These data align with prior studies associating the X allele with increased muscle susceptibility to damage and impaired regenerative capacity, factors that may translate into renal vulnerability in the context of systemic or local stressors [12, 13, 14, 15, 27, 28].

However, previous studies have demonstrated that the X allele is associated with increased susceptibility to muscle damage and reduced regenerative potential [12, 14, 15]. These characteristics may render carriers more vulnerable to renal damage in response to physiological stress [28]. For instance, individuals with the XX genotype are at greater risk of developing exercise‐induced rhabdomyolysis, a condition that can drive acute kidney injury [13]. Additionally, α‐actinin‐3 deficiency has been linked to altered responses to physical stress [29], which may cumulatively affect renal health over time.

To our knowledge, no previous studies have directly examined the relationship between the ACTN3 R577X polymorphism and CKD, particularly in patients undergoing HD. Although the polymorphism has been extensively studied in the context of athletic performance and skeletal muscle function, its role in renal disease has remained unexplored until now. Therefore, the present findings showing a higher frequency of the XX genotype among CKD patients and its association with earlier initiation of HD suggest that the ACTN3 R577X polymorphism may play a significant role in the pathogenesis of CKD. These results underscore the need for further research to elucidate the mechanisms underlying this association and to evaluate the potential of ACTN3 as a genetic marker for kidney disease progression risk.

To elucidate the role of Actn3 in the pathogenesis of renal failure, animal and in vitro experiments were performed. In both acute and chronic folic acid‐induced kidney injury models in mice, renal Actn3 expression was significantly elevated, even at 28 days post‐injury, when serum markers of renal function had normalized. This sustained upregulation indicates that ACTN3 may be part of a longer‐term cellular remodeling program associated with fibrotic progression rather than acute damage alone. The increased expression of fibrosis‐related genes (Col1a1, Acta2) in parallel with Actn3 further supports this hypothesis and provides a mechanistic basis linking ACTN3 to renal fibrogenesis. Importantly, our findings in primary mouse embryonic fibroblasts revealed that Actn3 is upregulated in response to TGF‐β, a key profibrotic cytokine [24]. This positions ACTN3 as a potential modulator of the myofibroblast phenotype. In contrast, Actn3 expression remained unchanged in fibroblasts stimulated with LPS, highlighting its specificity for fibrotic rather than inflammatory pathways. These experimental data reinforce the clinical findings and suggest that ACTN3 activation is tightly linked to fibrosis‐related signaling rather than a general stress response.

The AGT‐KO findings reinforce the concept that chronic fibrotic remodeling, independent of acute injury, engages Actn3 expression. By demonstrating that Actn3 upregulation is reproducible in both FA‐induced injury and spontaneous interstitial fibrosis, our results suggest that ACTN3 participates in a conserved profibrotic program [21]. These data strengthen the mechanistic plausibility that ACTN3 modulates cytoskeletal dynamics, myofibroblast activation, and ECM deposition in a fibrosis‐driven manner.

The dual role of ACTN3, as both a structural cytoskeletal protein and a modulator of tissue remodeling, may explain its implication in renal fibrosis. While the exact molecular mechanisms remain to be clarified, ACTN3 is known to influence actin filament dynamics, mechanical tension, and signal transduction pathways [17, 18], including those involving TGF‐β and angiotensin II, which are central to renal fibrogenesis [30]. Previous work has implicated these pathways in both muscle and cardiac fibrosis, supporting a potential mechanistic overlap in kidney tissue as well [5, 9].

In addition, ACTN3 is expressed in various non‐muscle cell types involved in renal homeostasis and fibrosis, including fibroblasts, T cells, and Henle's loop cells [10, 31, 32]. The enrichment of the X allele in patients with tubulointerstitial forms of kidney disease, a region where fibroblasts are abundant, further supports the hypothesis that ACTN3 may contribute to maladaptive repair responses in this compartment. It is plausible that ACTN3 deficiency contributes to renal injury through a combination of systemic (e.g., muscle injury‐induced nephrotoxicity) and local (e.g., fibroblast activation) mechanisms.

Interestingly, our findings that the R allele of ACTN3 is associated with delayed onset of hemodialysis in CKD patients are in agreement with previous studies in cardiovascular pathology [9]. Bernardez‐Pereira et al. [9] reported that patients with chronic heart failure carrying the ACTN3 R allele had significantly better long‐term survival compared to X allele carriers. This study suggests a cardioprotective effect of ACTN3 functional expression, which may be attributed to preserved structural integrity, enhanced resistance to stress‐induced damage, or modulation of fibrosis and inflammatory pathways [9]. Such parallels between the cardiac and renal systems are biologically plausible, as both organs rely on tightly regulated cytoskeletal and mechanical signaling pathways. Given that ACTN3 is involved in cytoskeletal dynamics [33], its deficiency could lead to maladaptive remodeling and fibrotic progression under chronic stress in multiple tissues, including the myocardium and renal interstitium [9]. These consistent observations support the hypothesis that the R allele confers resilience against organ failure, possibly through shared mechanisms of injury response, cytoskeletal stability, and control of fibroblast activation [9, 12, 13].

An intriguing aspect of our findings is the apparent paradox whereby both ACTN3 deficiency (via the R577X polymorphism) and renal injury independently contribute to unfavorable renal outcomes, while the injured kidney simultaneously upregulates Actn3 expression. One plausible explanation is that ACTN3 exerts a compensatory function in the renal response to injury, particularly through mechanisms involving fibroblast activation and cytoskeletal reorganization. It is important to emphasize that fibrosis is not exclusively pathological; rather, it constitutes a fundamental component of the kidney's attempt to repair tissue damage. In this context, the increased Actn3 expression observed in injured kidneys may reflect its participation in a reparative program aimed at preserving tissue integrity. Individuals carrying the functional R allele may benefit from ACTN3‐mediated regulation of cellular architecture and mechanotransduction, enabling more efficient and tightly controlled fibrotic remodeling. In contrast, individuals with the XX genotype may lack this regulatory capacity, predisposing them to disorganized or excessive fibrosis, particularly under repeated or sustained stress conditions. This impaired reparative potential may account for both the earlier onset of kidney failure and the higher prevalence of tubulointerstitial pathology observed among X allele carriers. Taken together, these findings suggest that ACTN3 may influence not only systemic stress responses (e.g., muscle damage) but also local renal repair mechanisms, with its absence shifting the balance toward maladaptive fibrosis.

This study has some limitations that should be acknowledged. First, the cross‐sectional design does not allow for causal inference between the ACTN3 R577X polymorphism and kidney disease progression. Second, although the association was observed in a well‐defined HD population, the lack of longitudinal follow‐up limits conclusions about the predictive value of ACTN3 genotyping over time. Third, while the experimental models support the biological plausibility of ACTN3 involvement in renal fibrosis, further functional studies are needed to elucidate the underlying molecular mechanisms. Lastly, the findings may not be generalizable to populations with different genetic backgrounds or CKD etiologies, and replication in independent cohorts is warranted.

Our findings suggest that ACTN3 genotyping may help identify individuals at higher risk for early progression to renal failure, particularly in the context of tubulointerstitial disease. In addition, ACTN3 expression may serve as a biomarker or therapeutic target in fibrotic kidney diseases. Genotyping for ACTN3 could potentially be integrated into early screening tools to improve risk stratification and guide patient monitoring. However, further studies are required to delineate its functional role, explore downstream signaling pathways, and determine whether ACTN3 interacts with other genetic variants that may influence renal outcomes.

## 5 Conclusion

This study provides the first evidence that the ACTN3 R577X polymorphism is associated with chronic kidney disease progression, particularly in patients undergoing hemodialysis. The higher frequency of the X allele in CKD patients, its association with significantly earlier initiation of dialysis, and the upregulation of *Actn3* expression in experimental models of renal injury collectively support a role for ACTN3 in kidney pathophysiology. The experimental data also demonstrate that ACTN3 is specifically upregulated in fibrotic, but not inflammatory, conditions, suggesting a mechanistic involvement in tissue remodeling and fibrosis. The inclusion of a second fibrosis model further supports the robustness and translational potential of the findings, strengthening the link between ACTN3 biology and renal fibrotic progression.

These findings point to ACTN3 as a potential genetic marker for early identification of individuals at risk for kidney failure, and possibly as a therapeutic target in fibrotic kidney diseases. Future studies are warranted to elucidate the molecular pathways linking ACTN3 function to renal fibrosis and to explore its clinical utility in risk stratification and personalized nephrology.

## Author Contributions

Conceptualization: C.C.B., R.B.O., M.B. Methodology: H.S.V., L.R.R., I.S., R.B.S., A.B., A.K.S., C.F.S., A.F.R. Investigation: R.B.S., H.S.V., L.R.R., I.S. Funding acquisition: R.C.A., A.S., C.C.B., M.B. Project administration: C.C.B. Supervision: C.C.B, M.B. Writing – original draft: C.C.B. Writing – review and editing: R.B.S., R.B.O., I.S., R.C.A., A.S., M.B., C.C.B.

## Funding

This work was supported by the Conselho Nacional de Desenvolvimento Científico e Tecnológico (CNPq)—Brasil (C.C.B.), Coordenação de Aperfeiçoamento de Pessoal de Nível Superior (CAPES)—Brasil (R.C.A., C.C.B.), and Fundação de Amparo à Pesquisa do Estado do Rio Grande do Sul (FAPERGS)—Brasil (A.S.).

## Conflicts of Interest

The authors declare no conflicts of interest.

## Data Availability

All data are available in the main text or the Supporting Information. Additional information may be requested from the corresponding author.
